# Giant Mesenteric Cyst With Gastric Perforation Masquerading As Obstructed Inguinal Hernia: A Rarest of the Rare Case

**DOI:** 10.7759/cureus.17919

**Published:** 2021-09-13

**Authors:** Manoj Joshua Lokavarapu, Farhanul Huda, Bhavaniprasad Mahindrakar, Shashank Kumar, Navin Kumar

**Affiliations:** 1 General Surgery, All India Institute of Medical Sciences, Rishikesh, Rishikesh, IND

**Keywords:** mesenteric cyst, inguinal hernia, gastric perforation, acute abdomen, intestinal obstruction

## Abstract

Mesenteric cysts are extremely rare intra-abdominal tumors. They usually present with an array of symptoms, usually non-specific, which leads to difficulty in diagnosing them. Occasionally these can present in the emergency as an acute abdomen. We report a rare presentation of a huge mesenteric cyst with gastric perforation, misdiagnosed clinically as obstructed inguinal hernia. A 50-year male presented with complaints of sudden severe pain in the abdomen along with swelling and pain in the right groin region with absolute constipation for the last 4 days. A clinical diagnosis of obstructed inguinal hernia was made. However, on radiological investigations, it was discovered as a giant intra-abdominal cyst herniating into the inguinal canal. On exploration, we were further surprised to find a concomitant gastric perforation.

In this case report, we highlight that mesenteric cysts can present as acute abdomen and, very rarely, can be associated with an accompanying cause of surgical abdomen.

## Introduction

Mesenteric cysts are extremely rare intra-abdominal tumors [1]. These are uncommon intra-abdominal tumors with an incidence of 1 in 27,000 to 1 in 250,000 [2]. They are more predominant in females than males and usually present in the fourth decade of life [3]. They can arise anywhere in the mesentery of the gastrointestinal tract, from the duodenum to the rectum. Most commonly, they are connected with the small bowel mesentery [3]. It was first described by Benevieni in 1507 [4]. They may present incidentally, insidiously, or as an acute life-threatening emergency [5]. Approximately 10% of patients with mesenteric and omental cysts present with an acute abdominal emergency [2]. The goal of treatment is the complete excision of the cyst by either resection or enucleation. At times, a portion of the cyst wall must be left in situ and marsupialized into the peritoneum and sclerosed [2]. The short- and long-term prognosis with mesenteric cysts are excellent, with a low recurrence rate, few complications related to treatment, and essentially no mortality [1]. Being a rare disease, minuscule information is available in the published literature. Herein, we outline a case of the huge mesenteric cyst with gastric perforation impersonating as obstructed inguinal hernia. To the best of our knowledge, no such case has been reported so far in English literature.

## Case presentation

**Figure 1:**
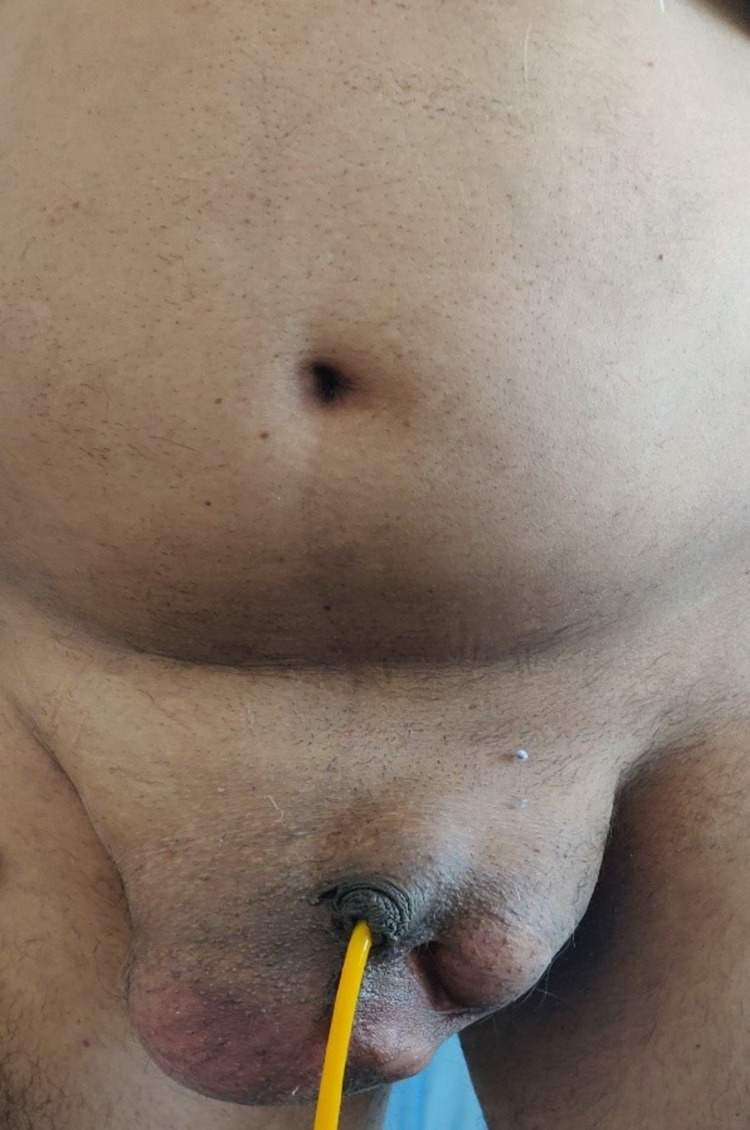
A tender swelling in the right inguinoscrotal region and the penis was seen buried inside the scrotum

A 50-year-old male came to the emergency room with complaints of sudden onset pain in the abdomen and over the swelling in the right groin for four days. The swelling in the right groin had been there for the last 18 months. The pain initially started at the swelling and later on became generalized to the whole abdomen. He also complained of absolute constipation. On examination patient’s vital signs were stable except for mild tachycardia and slightly raised body temperature. The patient had a distended abdomen with diffuse guarding all over. There was a tender swelling in the right inguinoscrotal region, and the penis was seen buried inside the scrotum (Figure 1). Laboratory reports such as complete hemogram, renal function test, and liver function test were within normal range

 

 

A clinical diagnosis of obstructed inguinal hernia was made with differentials being strangulated inguinal hernia with hollow viscus perforation. Ultrasound of the abdomen revealed an abdominal cyst of size 17cm X 8cm herniating into the right inguinal canal.

**Figure 2:**
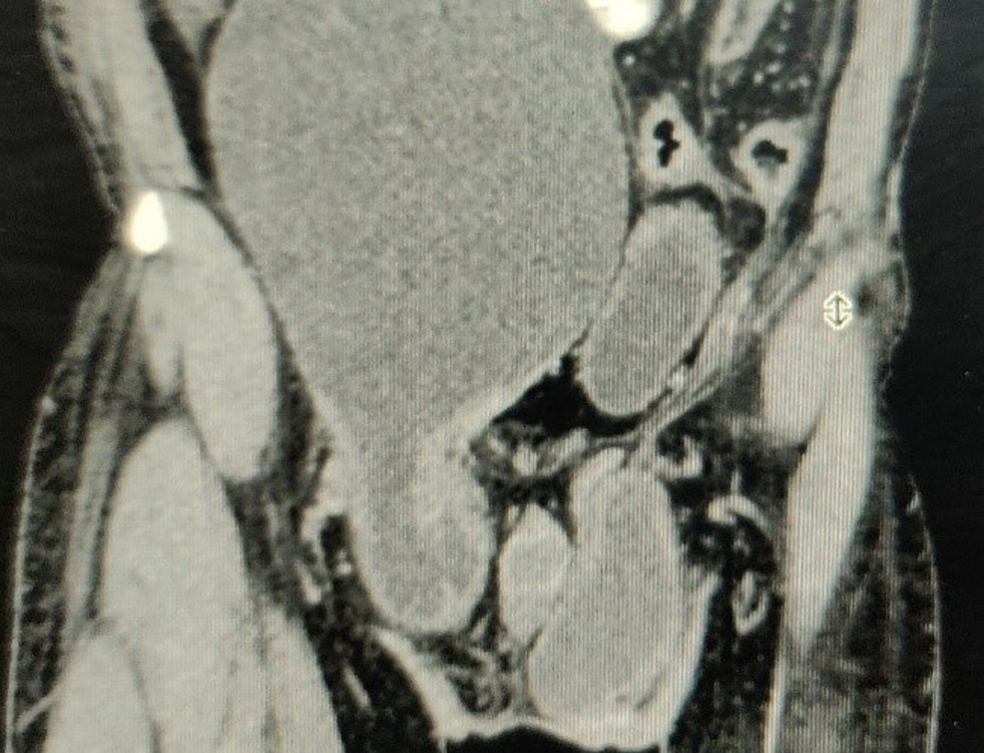
CECT abdomen showing a huge mesenteric cyst extending into the right inguinal region CECT - contrast-enhanced computerized tomography

A contrast-enhanced computerized tomography (CECT) of the abdomen was done, which showed a giant mesenteric cyst herniating into the right inguinal canal with minimal free fluid in the abdomen (Figure 2).

**Figure 3:**
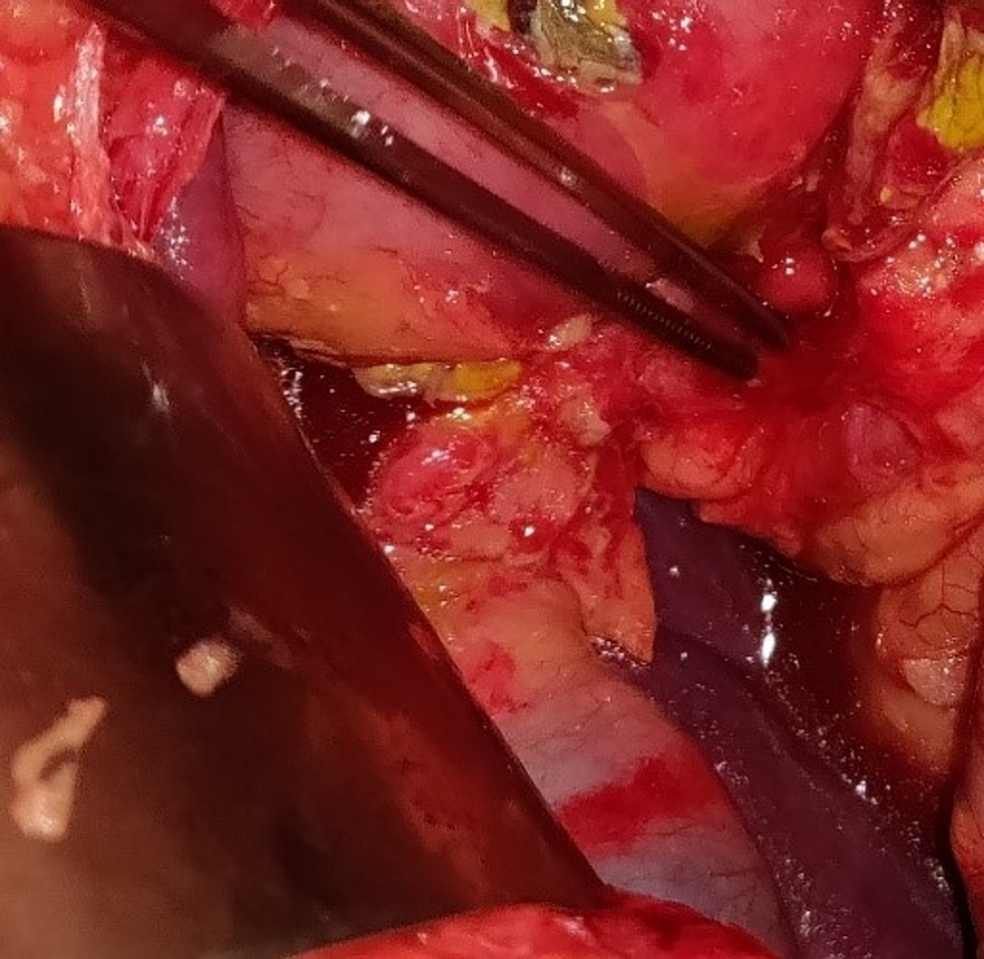
Prepyloric perforation

**Figure 4:**
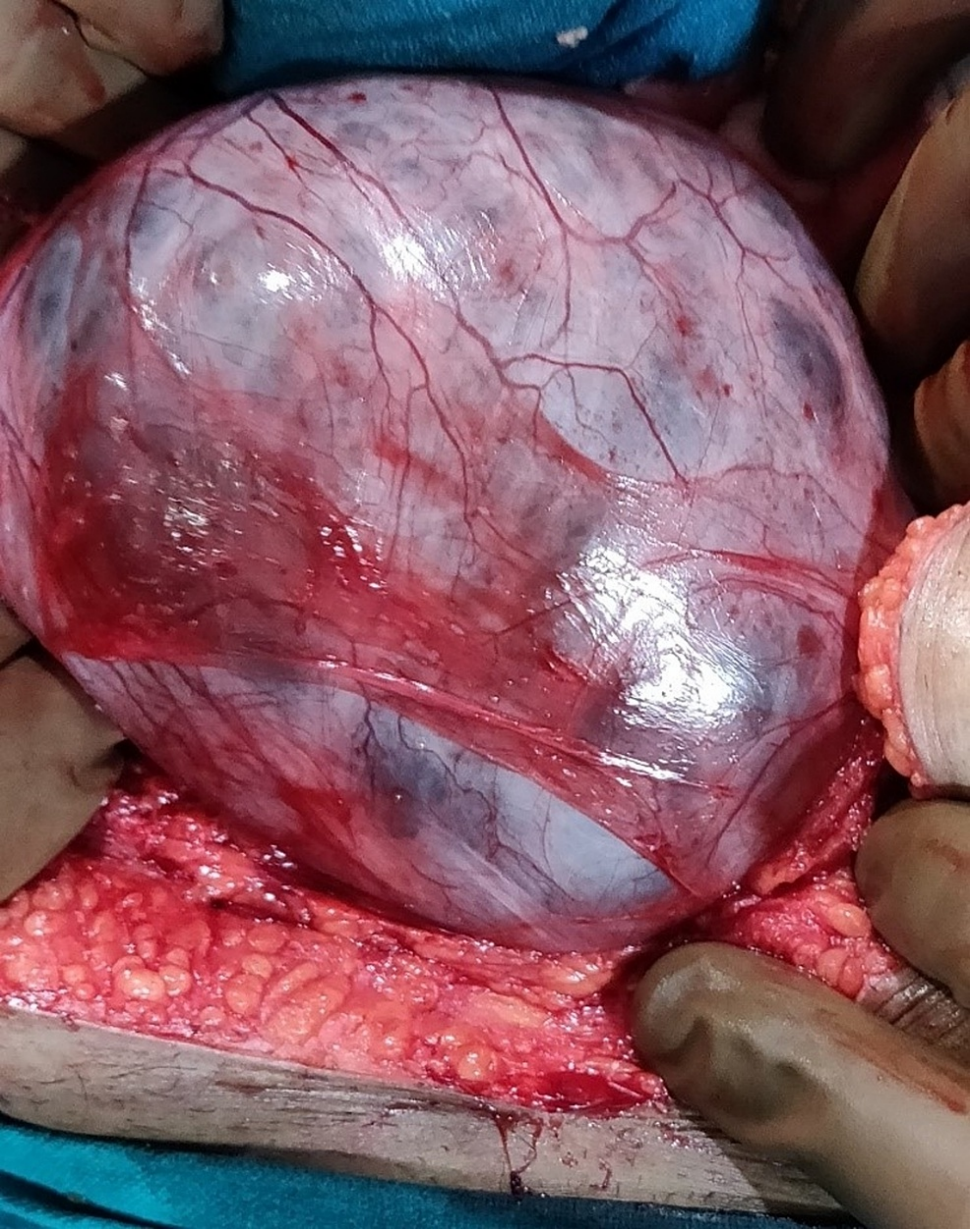
Intra-abdominal mesenteric cyst

**Figure 5:**
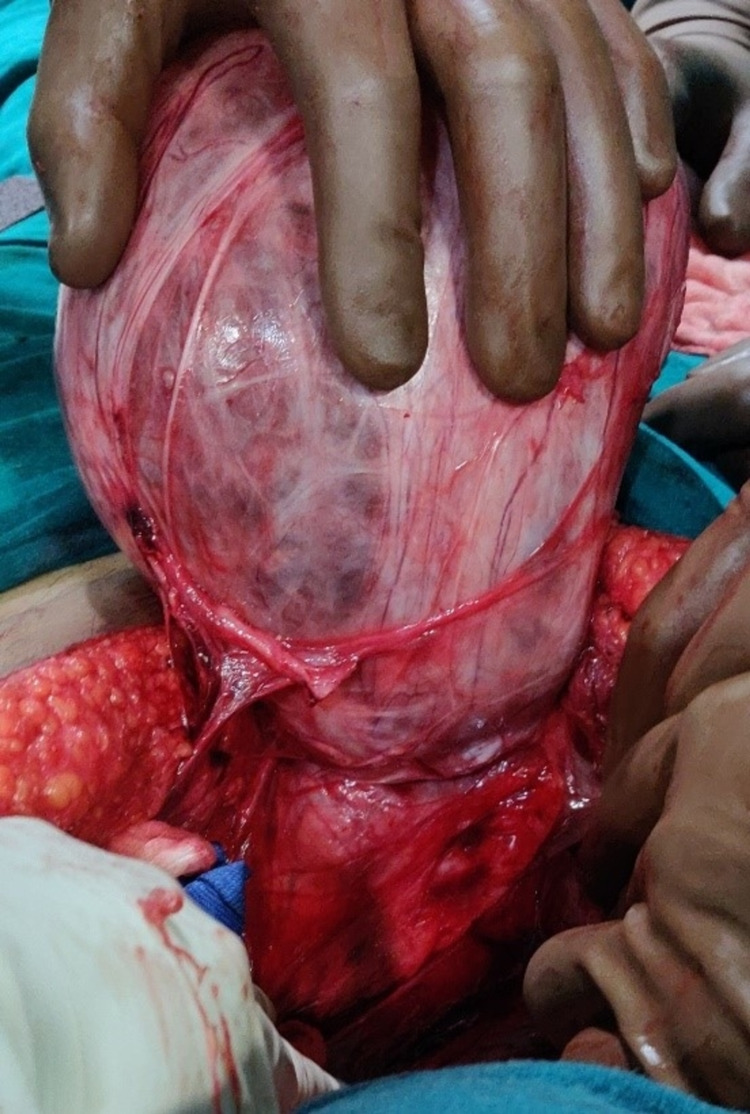
Mesenteric cyst herniating in the right groin region

**Figure 6:**
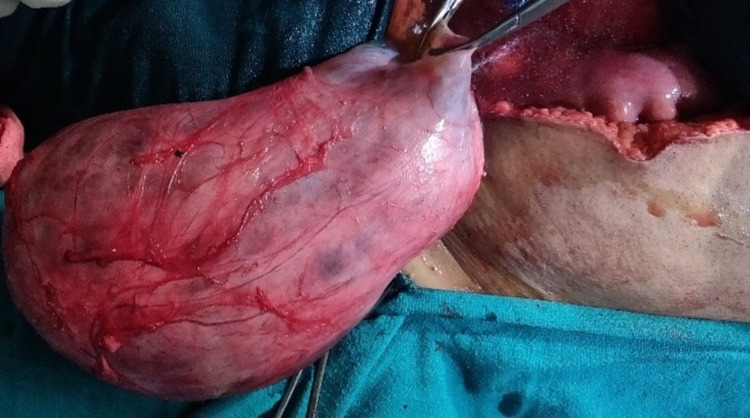
Enucleated Mesenteric cyst

The patient had to undergo exploratory laparotomy through a midline incision. We were surprised to find co-existing prepyloric perforation of 1 X 1cm along with a huge mesenteric cyst, which was occupying almost the whole of the abdomen and was seen herniating into the right inguinal region. The prepyloric perforation was repaired by the modified Graham’s omental patch repair technique, and complete enucleation of the mesenteric cyst was done (Figures 3-6).

 

 

 

 

 

 

The excised cyst was sent for histopathology, which showed fibro collagenous tissue lined with mesothelial lining compatible with a mesenteric cyst.

## Discussion

Mesenteric cysts are uncommon intra-abdominal tumors with an incidence of 1 in 27,000 to 1 in 250,000 [2]. They are more predominant in females than males and usually present in the fourth decade of life [2]. The exact etiology of these cysts remains unknown, and many theories for their evolution have been proposed. The widely accepted of these is that they are the consequence of the proliferation of ectopic lymphatics, which are present in the mesentery and have no communication with the lymphatic system [1,2,3].

Classically mesenteric cysts are classified into four types; 1) embryonic or developmental, 2) traumatic or acquired, 3) infective or degenerative, and 4) neoplastic [6]. Mesenteric cysts are also classified into six groups based on histopathological criteria. Out of these, malignant cystic mesothelioma has malignant potential and has a propensity to recur [6].

Kurtz et al. analyzed 162 cases of mesenteric cysts and described that the most common location of these cysts was in the small bowel mesentery [3]. These cysts have a wide spectrum of clinical features, from being completely asymptomatic to severely ill patients presenting as acute abdomen. The most common presentations are abdominal pain (58%) and distension (50%) in symptomatic individuals [7]. About one-third of these patients present as an acute abdominal emergency like intestinal obstruction, volvulus, or rupture of the cyst [7,8]. Out of these one-third patients with the acute presentation, two-third are pediatric patients. There are seven cases of mesenteric cysts presenting as inguinal hernia reported in the literature. Out of these, six were in children, and only one was in adults [9]. Our patient presented to the emergency department with diffuse abdominal pain, irreducible right inguinoscrotal swelling, and absolute constipation.

Mesenteric cysts presenting as strangulated umbilical and femoral hernia have also been reported in the literature [10]. A mesenteric cyst presenting as obstructed inguinal hernia in association with prepyloric perforation is extremely rare, as seen in our case.

Due to varied clinical presentations, preoperative diagnosis is almost impossible to make on a clinical basis. In our case, it was further complicated by the presence of concomitant gastric perforation, which led to clinical misdiagnosis. Although ultrasound is sensitive in detecting the cyst contents, a CT scan should be the first single initial investigation because it gives information about the location of the cyst and nature of contents. Precise diagnosis and treatment are necessary owing to the malignant transformation of cyst in 3% of cases [11]. In our case, the ultrasound showed an abdominal cyst herniating into the right inguinal canal. A contrast-enhanced CT scan confirmed the mesenteric cyst herniating into the right inguinal canal.

Surgical management with complete excision of the cyst is the treatment of choice to prevent recurrence and treat cyst-associated obstruction, volvulus, or herniation of the bowel. Bowel resection is necessary when the cyst is in the vicinity of the bowel or involving blood supply to the bowel. Total cystectomy by open approach is a method of choice. Laparoscopic excision of cyst has also been reported. The laparoscopic approach offers the advantage of early recovery and reduced hospital stay. In our case, we enucleated the mesenteric cyst and performed omental patch repair for perforation. In some cases, when the cyst is in the vicinity of vital structures marsupialization can also be done. This procedure is suboptimal and may require a second surgery in the future for recurrence [12].

## Conclusions

Mesenteric cysts are rare benign intra-abdominal lesions and large cysts are even less common. It can present acutely in the emergency and sometimes may be associated with a co-existing surgical emergency.
